# Analysis on external competency assessment for malaria microscopists in China

**DOI:** 10.1186/s12936-019-2996-3

**Published:** 2019-11-14

**Authors:** Mei Li, Hejun Zhou, He Yan, Jianhai Yin, Xinyu Feng, Zhigui Xia, Shuisen Zhou

**Affiliations:** 1 National Institute of Parasitic Diseases, Chinese Center for Diseases Control and Prevention, Shanghai, 200025 China; 2 Chinese Center for Tropical Diseases Research, Shanghai, 200025 China; 3 WHO Collaborating Centre for Tropical Diseases, Shanghai, 200025 China; 4 National Center for International Research on Tropical Diseases, Ministry of Science and Technology, Shanghai, 200025 China; 5 Key Laboratory of Parasite and Vector Biology, Ministry of Public Health, Shanghai, 200025 China

**Keywords:** WHO-ECAMM, Microscopist, Competency, Training course, PPS

## Abstract

**Background:**

In order to meet the requirement of malaria elimination (ME), three courses of the External Competency Assessment of Malaria Microscopists (ECAMM) were conducted during 2017–2018 in China by facilitators designated by the World Health Organization (WHO-ECAMM). A training course with a model copied from the WHO-ECAMM course was also held a week ahead of ECAMM in March 2018. Thirty-six participants completed these courses and obtained different results.

**Methods:**

The slide structures, agendas, score calculations, and the levels of certifications of the four courses strictly adhered to the WHO guidelines. All the data were collected in Excel 2016 and analysed in Graphpad Prism5 or SPSS 23. Significant differences were evaluated in Graphpad Prism5 by two-tailed paired t tests between the pre-assessment and final-assessment for each of the four courses, as well as one-way ANOVAs with Kruskal–Wallis tests and Dunn’s post hoc tests among the final assessments of the four courses. Correlations between participants’ competency results and their ages, years working on malaria, and numbers of malaria cases reported in their provinces were evaluated by bivariate correlations (two-tailed) and linear regression (excluding cases pairwise) in SPSS 23. The Pearson correlation coefficients (r values), P values (two tailed), adjusted R square (Adjusted R^2^), standardized coefficients (β) and Sig. P values were recorded. The percentages of participants who gave the right answer to each slide (PPS) in the final assessments of the three WHO-ECAMM courses were calculated. Correlation analysis between PPS and parasitaemia (100–2000 parasites/μL) of *Plasmodium falciparum* slides used in species identification and parasite counting, were also evaluated via bivariate correlations (two-tailed) tests.

**Results:**

Among the 36 participants, 16 participants were certificated as Level 1 (two from NRL), 10 were certified as Level 2 (one from NRL). Within the same course, participants had improved their average scores from pre-assessments to final assessments. The numbers of malaria cases reported in participants’ provinces were strongly correlated to their species identification (SI) scores; r = 0.45, P = 0.040, n = 21; r = 0.57, P = 0.001, n = 32; r = 0.56, P = 0.007). The parasitaemia of *P. falciparum* within 100–2000 parasites/μL was correlated significantly (r = 0.44, P = 0.008, n = 36) with the PPS of all counting slides but not with slides for identification (r = − 0.018, P = 0.93, n = 30).

**Conclusions:**

The analysis and comparison of participants’ competency results not only verified that the model of the WHO-ECAMM course had strong power in improving and assessing microscopists’ competencies but also reflected the correlation between decreased numbers of indigenous malaria cases and microscopists’ competencies in certain areas in China.

## Background

China is planning to declare malaria elimination (ME) in 2020 [1]. To support the declaration and sustain the achievements in ME, a sensitive surveillance system based on precise and timely diagnosis of malaria cases in current and future China is required [2–7]. Hence, certificated microscopists with high levels of competency in malaria diagnosis were essential. In the past dozen years, the model for the External Competency Assessment of Malaria Microscopists (ECAMM) courses—developed by the World Health Organization (WHO)—has been conducted in more than 100 selected countries in the West Pacific Region, Southeast Asia Region, Africa Region and Eastern Mediterranean Region. This model had been verified to be an effective model to evaluate competencies of microscopists. In order to meet the requirement of ME, three WHO-ECAMM courses were held during 2017–2018, which were attended by 36 Chinese microscopists from either the National Reference Laboratory for Malaria Diagnosis (NRL) or 31 provincial laboratories. To obtain preliminary knowledge prior to the WHO-ECAMM course, 12 microscopists from the course in March 2018 received a training course with a model copied from the WHO-ECAMM course. The 36 participants had different backgrounds and obtained different results from the WHO-ECAMM courses. In this study, the competency results from these four courses were reported and analysed to determin ways to best sustain and enhance these competencies in China.

## Methods

### Administration of WHO-ECAMM and the training courses

WHO-ECAMM courses were held at the National Institute of Parasitic Diseases (NIPD) at the Chinese Center for Disease Control and Prevention (China CDC) in Shanghai by the facilitator, Ken Lilley (from WHO), in September 2017, March 2018 and November 2018. The slide structure and agenda strictly followed WHO guidelines [8, 9]. The training course was conducted a week ahead of the WHO-ECAMM course that was held in March 2018. The slide structure (Table 1), agenda, score calculations and level of certification of the training course were copied from WHO-ECAMM [8, 9], in which all participants were required to read 74 slides in 4 days, of which 56 slides were used for competency assessment. The slides for the four courses were all from the slide banks of the Research Institute for Tropical Medicine (RITM). Participants’ scores or accuracy (%) for parasite detection (PD), species identification (SI), and parasite counting (PC) were calculated by the following three formulas:

$${\text{PD}} = {\text{Pp}} \times 100/\left( {Pp + FNp} \right) ; {\text{ SI}} = {\text{Np}} \times 100/\left( {Np + FPp} \right);{\text{ and PC}} = {\text{CCs}} \times 100/{\text{T}} .$$

**Table 1:** Slides structure in training course

	Parasitemia (P/μL)	Species identification						Parasite counting
		Pf	Pv	Pm	Po	Mix	Neg	
Slides in final assessment	100–200	2	–	–	–	–	20	1
	200–500	6	2	–	–	–		5
	500–2000	2	2	2	1	–		6
	2000–5000	–	–	–	1	4		–
	≥ 10,000	–	–	–	–	–		2
	Total	10	4	2	2	4		14
		42						14
Slides in pre-assessment	100–200		–	–	–	–	5	1
	200–500	2	2	–	–	–		1
	500–2000	1	–	1	–	–		2
	2000–5000	–	–	–	1	1		–
	≥ 10,000	–	–	–	–	–		1
	Total	3	2	1	1	1	5	5
		13						5

Pf, *Plasmodium falciparum*; Pv, *Plasmodium vivax*; Pm, *Plasmodium malaria*; Po, *Plasmodium ovale*; Neg, no malaria parasite present

Pp denotes the number of slides with malaria parasites (positives slides) noted by the participant. FNp denotes the number of false negatives noted by the participant. Np denotes the number of negatives noted by the participant. FPp denotes the number of false positives noted by the participant. CCs denotes the number of slides with PC within 25% of the true count noted by the participant. T denotes the total number used for assessment. A participant was certificated as having a high level (1 or 2 levels) of competency only when a participant’s score of PD and SI were both equal to or higher than 80% and their PC score was equal to or higher than 40% (Table 2).

**Table 2:** Competency levels and criteria

Competency level	PD (%)	SI (%)	PC (%)
1	≥ 90	≥ 90	≥ 50
2	80 to < 90	80 to < 90	40 to < 50
3	70 to < 80	70 to < 80	30 to < 40
4	< 70	< 70	< 30

PD, participants’ scores or accuracy (%) for parasite detection; SI, participants’ scores or accuracy (%) for species identification; PC, participants’ scores or accuracy (%) for parasite counting

### Background of participants from the three different WHO-ECAMM courses

For the 12 participants in the WHO-ECAMM course in March 2018, seven of whom came from non-malaria-epidemic provinces, one participant had attended the WHO-ECAMM course in the past and all received the training a week ahead of the main course. Twenty-four participants in the other two WHO-ECAMM courses were from NRL and malaria-epidemic provinces. Nine of the 12 participants attending the course in November 2018 had taken part in the WHO-ECAMM course at least once in the past three to 6 years. However, most of the participants (11/12) from the course in September 2017 had neither of the above experiences.

### Data collection

All the data were collected in Excel 2016. Detailed basic information of the 36 participants (e.g., ages and years working on malaria) from the three WHO-ECAMM courses and their competency results—including PD, SI, PC and certification levels (CLs)—were collected and consulted from mission reports of the WHO-ECAMM facilitator. The malaria situations (reported cases) in 2014–2017 in the 31 provinces from which the 32 participants worked were gathered (two participants from same province) [10–13]. Also, the answer of each participant given to each slide in the final assessment of the three WHO-ECAMM courses was recorded.

### Data analysis

All the data were analysed in Graphpad Prism5 or SPSS 23. To compare the competency results (mean values with 95% confidence interval, CI) between the pre-assessments and final-assessments from each of the four courses, significant differences (P < 0.05, significant; P ≥ 0.05, not significant) were evaluated by two-tailed paired t tests in Graphpad Prism5. Additionally, the final-assessments of the four courses were evaluated by one-way ANOVAs with Kruskal–Wallis tests and Dunn’s post hoc tests in Graphpad Prism5. The Pearson correlation coefficients (r values) and P values (two tailed) among the 36 participants’ competency results of PD, SI and PC scores were evaluated by bivariate correlations (two-tailed) in SPSS 23. To verify the factors influencing participants’ competency results, the correlations between participants’ competency results and their ages, years working on malaria, and numbers of malaria cases reported in their provinces were also evaluated. Firstly, the 36 participants’ ages, years working on malaria, and the numbers of malaria cases reported in the 31 provinces in the different courses were counted and compared. The correlation analysis was carried out within each of the four combinations that were assembled by data in the final assessments of the four courses by bivariate correlations and linear regression in SPSS 23. The correlation coefficients (r values), P values (two tailed), adjusted R square (Adjusted R^2^), standardized coefficients (β) and Sig. P values were recorded. Combination 1 included data in the WHO-ECAMM course in September 2017 and in the training course in which participants attended prior to the main course. Combination 2 included data of Combination 1 and those in the WHO-ECAMM course in November 2018. Data for Combinations 3 and 4 were all obtained from the WHO-ECAMM courses. Combination 3 included data from September 2017 and November 2018 in which participants had not received similar training over the past 3 years. Combination 4 included data from the three WHO-ECAMM courses.

The percentages of participants who gave the right answer to each slide (PPS) in the final assessment of the three WHO-ECAMM courses were calculated and recorded.

Pearson correlation coefficients (r values) and P values (two tailed) between the PPS and parasitaemia (100–2000 parasites/μL) of the *Plasmodium falciparum* slides used in species identification and parasite counting were evaluated by bivariate correlations (two-tailed) test separately.

## Results

### Competency results in the training course

The competency results in the pre-assessment and final-assessment were shown in Table 3. One participant from NRL was certified at Level 1, four participants from provincial laboratories were certificated as Level 2, and the other participants were certified as either Level 3 or Level 4. All participants improved their scores. The average scores improved as follows: PD increased from 92% (CI 84–100%) to 93% (CI 88–97%); PC increased from 28% (CI 16–41%) to 42% (CI 31–54%); and SI increased significantly from 58% (CI 40–76%) to 77% (CI 69–86%; P = 0.007, n = 12, paired *t* test).

**Table 3:** Competency results of 12 participants in training course

Participants	Codes	PD (%)		SI (%)^a^		PC (%)		TCL
		Pre-assessment	Final assessment	Pre-assessment	Final assessment	Pre-assessment	Final assessment	
Competency results	12	100	95	62	77	20	43	3
	13	100	95	96	87	20	57	2
	14	100	100	69	86	20	64	2
	N	75	90	23	50	40	14	4
	15	100	86	65	79	0	36	3
	16	100	95	58	69	40	21	4
	17	100	100	88	88	20	43	2
	N	100	95	100	93	60	50	1
	18	88	95	58	87	60	79	2
	19	63	76	31	60	0	29	4
	20	88	90	23	70	20	36	3
	21	88	95	23	82	40	36	3
Average		92	93	58	77	28	42	–
95% confidence interval								
Lower		84	8	40	69	16	31	–
Upper		100	97	76	86	41	54	

PD, participants’ scores or accuracy (%) for parasite detection; SI, participants’ scores or accuracy (%) for species identification; PC, participants’ scores or accuracy (%) for parasite counting; TCL, competency level in training course; Pre, pre assessment; Fin, final assessment; Codes, each code represented one province; N, National Institute of Parasitic Diseases

^a^The scores increasing great significantly at 0.01 level

### Competency results of the three WHO-ECAMM courses

The competency results of all participants in the three WHO-ECAMM courses were shown in Table 4. In total, 16 participants were certificated as Level 1 (two from NRL), 10 participants were certified as Level 2 (one from NRL), and the others were certified as Level 3 or Level 4. Separately, in the course from September 2017, two participants were certificated as Level 1, four participants were certified as Level 2, three participants were certified as Level 3, and three participants were certified as Level 4. In the course from March 2018, six participants were certificated as Level 1, five participants were certified as Level 2, and one participant was certified as Level 4. In the course from November 2018, eight participants were certificated as Level 1, one participant was certified as Level 2, and three participants were certified as Level 3.

**Table 4:** Competency results of 36 participants in WHO-ECAMM courses

Courses	Codes	Course of Sep 2017							Codes	Course of Mar 2018							Codes	Course of Nov 2018						
		PD (%)		SI (%)^b^		PC (%)^b^		CL		PD (%)		SI (%)^b^		PC (%)		CL		PD (%)^b^		SI (%)^a^		PC (%)		CL
		Pre	Fin	Pre	Fin	Pre	Fin			Pre	Fin	Pre	Fin	Pre	Fin			Pre	Fin	Pre	Fin	Pre	Fin	
Competency results	1	100	100	73	95	40	71	1	12	100	100	100	98	60	50	1	22	75	100	69	92	60	36	3
	2	100	95	92	88	0	71	2	13	100	95	92	93	40	43	2	13	100	100	85	94	60	36	3
	3	100	100	88	82	60	43	2	14	88	100	81	100	60	29	4	23	100	100	77	96	20	50	1
	4	100	95	77	86	20	43	2	N	100	100	73	85	20	50	2	24	88	100	88	100	20	57	1
	5	100	95	77	89	40	36	3	15	88	100	85	88	20	64	2	25	100	100	92	94	60	57	1
	N	88	95	81	92	40	36	3	16	88	100	81	94	40	64	1	26	88	100	73	98	60	71	1
	6	100	100	81	81	0	14	4	17	100	100	96	93	40	64	1	27	88	100	92	96	80	57	1
	7	100	91	73	96	40	50	1	N	100	100	92	100	40	86	1	28	88	100	77	96	20	50	1
	8	88	100	19	73	20	21	4	18	100	100	100	94	40	64	1	29	100	100	96	93	20	50	1
	9	100	100	77	86	0	43	2	19	100	100	85	92	60	71	1	30	100	86	31	82	20	36	3
	10	100	100	65	76	20	57	3	20	100	100	85	94	80	43	2	31	88	100	88	98	80	50	1
	11	100	95	54	74	20	21	4	21	100	100	73	98	40	43	2	N	88	95	92	92	60	57	1
Average		98	97	71	85	25	42	–		97	100	87	94	45	56	–		92	98	80	94	47	50	–
95% confidence interval																								
Lower		95	95	59	80	13	31	–		94	99	81	91	34	46	–		87	96	69	91	31	43	–
Upper		101	99	84	90	37	54	–		100	101	93	97	56	66	–		97	101	91	97	62	57	–

PD, participants’ scores or accuracy (%) for parasite detection; SI, participants’ scores or accuracy (%) for species identification; PC, participants’ scores or accuracy (%) for parasite counting; Pre, pre assessment; Fin, final assessment; CL, competency levels; Codes, each code represent one province; N, National Institute of Parasitic diseases

^a^The scores increasing great significantly from pre-assessment to final assessment, P ≤ 0.01

^b^The scores increasing significantly from pre-assessment to final assessment, 0.01 < P < 0.05

Correlation analysis showed that the 36 participants’ competencies of SI were significantly correlated with PC (r = 0.48, P = 0.003; n = 36). All average scores of the 36 participants in the final assessments had improved compared with those in pre-assessments, except for the average PD score in September 2017, which was 98% (CI 95–101%) and 97% (CI 95–99%; P = 0.63, n = 12), respectively. Also, participants from September 2017 had improved their SI and PC scores significantly, which increased from 71% (CI 59–84%) to 85% (CI 80–90%; P = 0.014, n = 12), and from 25% (CI 13–37%) to 42% (CI 31–54%; P = 0.036; n = 12), respectively. The participants from March 2018 improved their SI score significantly from 87% (CI 81–93%) to 94% (CI 91–97%; P = 0.021, n = 12). Participants from November 2018 improved both their PD and SI scores significantly from 92% (CI 87–97%) to 98% (CI 96–101%; P = 0.044, n = 12), and from 80% (CI 69–91%) to 94% (CI 91–97%) (P = 0.0065), respectively.

Among the three courses, participants’ average PD scores in the final assessments were 97% (CI 95–99%), 100% (CI 99–101%) and 98% (CI 96–101%). Following a one-way ANOVA with a Kruskal–Wallis test, there was no significant difference among these scores (P = 0.059; n = 12, 12, 12). For the average scores of SI in the three final assessments, participants from the three courses obtained SIs of 85%, 94% and 94%. Participants’ average SI scores in the two courses from 2018 were significantly higher than that in September 2017 (P = 0.0034, Kruskal–Wallis test; n = 12, 12, 12), but there was no significant difference between the courses during 2018. For the average score of PC in the final assessments, participants from the three courses obtained PC scores of 42% (CI 31–54%), 56% (CI 46–66%) and 50% (CI 43–57%). There were no significant differences among these scores (Kruskal–Wallis test, n = 12, 12, 12).

Among the four courses, the competency results in the final assessments of the training course were the lowest. However, analysis showed that 12 participants in this course improved their average scores of PD, SI or PC (P = 0.0084, P = 0.0003, P = 0.0043; Kruskal–Wallis test; n = 12, 12, 12, 12) from pre-assessment in the training course to the final-assessment in the WHO-ECAMM course in March 2018.

### Influence of parasitaemia on participants’ competency results of *P. falciparum* identification and counting

The correlation analysis showed that there was no correlation between the PPS and parasitaemia of *P. falciparum* slides (r = − 0.018, P = 0.93, n = 30) that were used in species identification. However, these two datasets correlated significantly (r = 0.44, P = 0.008, n = 36) when the slides were used for parasite counting and their parasitaemia were within 100–2000 parasites/μL.

### Analysis of participants’ backgrounds and basic information

The backgrounds and basic information of the 36 participants from 31 provincial laboratories and NRL in the different courses were analysed and compared (Table 5, 6). The average age of participants in the three WHO-ECAMM courses was 38 years old (CI 36–40 years old, n = 36). Among them, the youngest and oldest participants were 28 and 54 years old. There was no significant difference among the ages of participants from the three different WHO-ECAMM courses (P = 0.61). All of the participants had experience working on malaria. They had worked on malaria for at least 1 year and for as long as 32 years. On average, they had worked in malaria diagnosis for 12 years (CI 9–14 years, n = 36). The numbers of malaria cases in the 31 provinces ranged from 1 to 469 (Mean number = 101 cases, CI 59–144 cases, n = 31). Participants from provinces where more cases were reported tended to work on malaria longer (r = 0.37, P = 0.036). There were no variations in their ages in terms of the differences in the numbers of malaria cases among their different provinces (r = 0.21, P = 0.25).

**Table 5:** Basic information of all participants in 3 WHO-ECAMM courses

Sep 2017				Mar 2018				Nov 2018			
Codes^a^	Ages	Years	Cases	Codes^a^	Ages	Years	Cases	Codes^a^	Ages	Years	Cases
1	43	18	215	12	32	3	3	22	35	10	109
2	36	14	126	13	32	6	327	13	30	2	327
3	34	8	11	14	49	5	10	23	39	13	207
4	54	32	172	N^b^	40	10	–	24	48	28	469
5	51	30	69	15	40	16	18	25	35	8	192
N^b^	32	6	–	16	33	5	53	26	37	12	26
6	44	17	30	17	33	6	32	27	34	13	125
7	40	11	15	N^b^	33	7	–	28	50	30	273
8	32	5	5	18	39	7	81	29	37	13	277
9	43	19	55	19	36	1	5	30	43	13	132
10	32	11	44	20	38	1	5	31	36	13	49
11	39	18	4	21	28	1	1	N^b^	42	8	–
n^c^	12	12	11	n	12	12	10	n	12	12	11
Average	40	16	68	17	36	6	54	25	39	14	199
95% confidence interval											
Lower	35	10	19	14	33	3	− 18	22	35	9	111
Upper	45	21	116	19	40	8	125	29	43	19	287

^a^Each code represented one province. The codes were numbered randomly; N, National Institute of Parasitic Diseases; n, number of values

**Table 6:** Basic information of all participants in 4 combinations

	Combination 1			Combination 2			Combination 3			Combination 4		
	Ages	Years	Cases	Ages	Yearns	Cases	Ages	Years	Cases	Ages	Yearns	Cases
Sep 2017	×	×	×	×	×	×	×	×	×	×	×	×
Mar 2018	×	×	×	×	×	×	O	O	O	×	×	×
Nov 2018	O	O	O	×	×	×	×	×	×	×	×	×
No. of values	24	24	21^a^	36	36	32^ab^	24	24	22	36	36	32^ab^
Average	38	11	61	38	12	108	39	15	133	38	12	108
95% confidence interval												
Lower	35	7	23	36	9	65	37	11	79	36	9	65
Upper	40	14	99	40	14	152	42	18	188	40	14	152

×, data in this course were applied in analysis; O, data in this course were not applied in analysis

^a^Because there were no data for participants from NRL

^b^Because two participants came from same province

Participants from the WHO-ECAMM course of March 2018 had worked in malaria diagnosis significantly less time (mean = 6 years, CI 3–8 years, n = 12) than those from the other two courses (mean = 16 years, CI 10–21 years, n = 12; mean = 14 years, CI 9–19 years, n = 12; P = 0.0040, Kruskal–Wallis test). The average number of malaria cases (Mean = 199 cases, CI 111–289 cases, n = 11) in provinces from participants in the course of November 2018 was significantly greater than those in provinces from participants in the other two courses (mean = 68 cases, CI 19–116 cases, n = 11; mean = 54 cases, CI − 20 to 116 cases, n = 10; P = 0.005, Kruskal–Wallis test).

### Evaluation of correlation between competency results and factors

The correlation analysis results of data in the four combinations were shown in Table 7. Nearly none of the combinations showed a correlation of participants’ competency results with their ages and years that participants worked on malaria.

**Table 7:** Correlation values of 3 factors and competency results among 4 combinations

Factors	Statistics	Values	Combinations											
			Combination 1^a^			Combination 2^a^			Combination 3			Combination 4		
			PD	SI	PC	PD	SI	PC	PD	SI	PC	PD	SI	PC
Ages	Pearson correlations	r	0.38	0.16	0.068	− 0.17	0.15	0.096	− 0.26	0.12	− 0.014	− 0.24	0.013	0.17
		P	0.86	0.45	0.75	0.92	0.40	0.58	0.22	0.58	0.95	0.16	0.94	0.33
	Regression coefficients	Adjusted R^2^	0.094	0.096	0.15	0.075	0.28	0.013	0.17	0.20	0.037	0.043	0.13	0.049
		β	− 0.24	− 0.069	0.26	− 0.43	− 0.19	0.14	− 1.16	0.15	− 0.32	− 0.23	0.036	− 0.24
		Sig. P	0.51	0.84	0.43	0.14	0.46	0.63	0.026*	0.75	0.56	0.45	0.20	0.43
Yeas	Pearson correlations	r	0.19	0.29	0.005	0.21	0.33	0.089	0.063	0.10	0.045	− 0.19	− 0.14	− 0.081
		P	0.37	0.17	0.98	0.22	0.052	0.60	0.77	0.64	0.83	0.27	0.41	0.64
	Regression coefficients	Adjusted R^2^	0.094	0.096	0.15	0.075	0.28	0.013	0.17	0.20	0.037	0.043	0.13	0.049
		β	0.35	0.22	− 0.36	0.47	0.29	− 0.15	0.92	− 0.19	0.25	− 0.019	− 0.59	0.053
		Sig. P	0.37	0.53	0.29	0.12	0.28	0.63	0.071	0.69	0.65	0.95	0.051	0.87
Cases	Pearson correlations	r	0.15	0.45	0.47	0.29	0.57	0.32	0.20	0.56	0.30	− 022	0.30	0.12
		P	0.52	0.040*	0.030*	0.11	0.001**	0.078	0.38	0.007**	0.17	0.90	0.092	0.53
	Regression coefficients	Adjusted R^2^	0.094	0.096	0.15	0.075	0.28	0.013	0.17	0.20	0.037	0.043	0.13	0.049
		β	0.079	0.39	0.55	0.20	0.50	0.34	1.11	0.57	0.31	0.033	0.45	0.15
		Sig. P	0.75	0.10	0.023*	0.29	0.006**	0.089	0.28	0.011*	0.29	0.87	0.021*	0.47

Combination 1: In this combination, the competency results were obtained by participants when they firstly took part in such assessment as WHO-ECAMM courses

Combination 2: In this combination, the competency results were obtained by participants when they had not implemented such practical operation as WHO-ECAMM courses recently

Combination 3: In this combination, the competency results were obtained by participants in WHO-ECAMM courses when they had not implemented such practical operation as WHO-ECAMM courses recently

Combination 4: In this combination, the competency results were obtained by participants in WHO-ECAMM courses with one-third of them had implemented such practical operation as WHO-ECAMM courses recently

PD, participants’ scores or accuracy (%) for parasite detection; SI, participants’ scores or accuracy (%) for species identification; PC, participants’ scores or accuracy (%) for parasite counting

^a^Competency results in training course instead of those in WHO-ECAMM course were applied

** Correction is significant at 0.01 level

* Correction is significant at 0.05 level

For the numbers of malaria cases in provinces, the correlation analysis between them and participants’ SI scores presented a significant positive correlation in Combinations 1, 2 and 3 (r = 0.45, P = 0.040, n = 21; r = 0.57, P = 0.001, n = 32; r = 0.56, P = 0.007, n = 22). The regression results were also well supported these correlations in Combination 2 and 3 (Adjusted R^2^ = 0.28, 0.20; β = 0.50, 0.57; Sig. P = 0.006, 0.011; n = 32, 22). However, the correlation disappeared or degraded in Combination 4 (r = 0.30, P = 0.092, Adjusted R^2^ = 0.13; β = 0.45; Sig. P = 0.021; n = 32). In all correlation analysis, no factors were shown to correlate with participants’ PD scores (|r| ≤ 0.38, P ≥ 0.11).

## Discussion and conclusion

In 2017, no indigenous cases of malaria were reported in China, which gave a boost to all Chinese people [10, 14]. This represented a crucial milestone for China in terms of reaching the ME goal. However, it should be noted that imported cases were still reported in all 31 provinces in China, including seven non-epidemic provinces [5, 6, 10–13]. Additionally, the malaria patients were not restricted to known areas or designated by natural environments any longer. If the cases could not be found in time, especially in some areas where malaria cases were seldom or had not been reported for a long time, severe cases (even potentially fatal cases) [5, 6, 10–13] and/or retransmission could occur [15–17]. Hence, continued efforts were needed to strengthen the malaria monitoring system, which depends on a strong and widely distributed laboratory network that can ensure accurate and timely diagnoses of malaria cases.

From September 2017 to the end of November 2018, three WHO-ECAMM courses were held to determine microscopists’ competency levels and to strengthen the laboratory network for malaria diagnosis in China. Thirty-six participants were certificated in these courses and their certifications were valid until 2020. Thirty-two of them covered all provinces in China. Most of them were young and experienced. Sixteen participants (including two from NRL) were certificated as Level 1 and 10 participants (including one from NRL) were certified as Level 2. All of them were either trainers, facilitators, or inspectors in the laboratory network of malaria diagnosis for achieving the ME goal in China. However, because malaria activities were organized in provincial units under a national level in China, at least one microscopist with a high level (1 or 2 level) in each province was essential for the laboratory network. Thus, 26 microscopists with high levels is not sufficient for such a large area of China. Hence, more training courses and WHO-ECAMM courses were required to strengthen and certify microscopists’ competencies.

Analysis on the competency results of the participants showed that there were differences among the three WHO-ECAMM courses, especially in terms of SI and PD scores. PD and SI were the key requirements in the accurate diagnosis of malaria. These two competencies not only influenced each other, but also influenced PC competency greatly. As such, it was necessary to find the factors that might influence these two competencies. Thus participants’ basic information and backgrounds were investigated. The competency results in the training course were also applied in this investigation because the results reflected their original competencies in their routine work. The results showed that (data in Combination 1, 2 and 3) if there were no training the numbers of malaria cases reported in participants’ provinces significantly correlated to participants’ SI scores. In contrast, competencies of even experienced microscopists in provinces where malaria gradually became a rare disease concomitantly decreased because they did not have enough malaria slides to review in their routine work over a long period of time [5, 6, 10–13, 18]. Additionally, microscopists in provinces where malaria cases were less reported usually would not remain at their posts for a long time, and new staff in these provinces would not be able to improve their competencies because they would have less of a chance of reading slides [4, 18–21]. These outcomes were also roughly in accordance with the trend of participants’ competency results in different courses and their backgrounds and/or basic information.

Surprisingly, although their previous competency results in the training course were worse, participants’ results in the WHO-ECAMM course of March 2018 were distinctly better than those in 2017 and were not inferior to those in November 2018. This significant change was ascribed to the training course held ahead of the WHO-ECAMM course. The training course had a model that was copied from the WHO-ECAMM and it improved these participants’ competencies greatly and led to the disappearance or degradation of the influence of participants’ previous working experiences (Combination 4). This power was consistent with the significant improvement of participants’ assessment results from pre-assessment to final assessment in each course.

Because of the strong power of the model of the WHO-ECAMM course in improving and assessing microscopists’ competencies, this model provides a new perspective for simultaneously training and assessing microscopists in China, especially in areas where malaria cases have been occasional or seldom reported in recent years. In these areas, microscopists usually had worked for a period but their competency could not sustain or improve due to the nature of their routine work. Even slides might be inadequate for conducting training courses in some provinces. As such, this kind of model (WHO-ECAMM) might not be applicable in this case.

Besides participants’ experiences, the slides themselves also influenced their competency results. Extensive misidentification of *P. falciparum* was not regarded as a difficult point for all microscopists but only for those who had less of a chance to examine the slides. However, parasite counting was difficult for all microscopists, especially when counting slides with low parasitaemia (one less or more parasite counted might influence the results greatly). Additionally, according to the analysis, participants’ PC competencies were significantly influenced by their SI competencies. Hence, microscopists’ capabilities both on SI should be strengthened before making improvements in PC competency. Additionally, counting skills that strictly followed the rules recommended by WHO were also practical for accurate counting. All participants in the course of March 2018 and nine participants in the course of November 2018 had experienced formal counting practice repeatedly. Consequently, they mastered their counting skills more skillfully than those in the course of September 2017, in which no participants had this experience. However, the low score or PPS in counting also indicated that most of the participants in China seldom counted parasites in their routine work.

## Data Availability

The data and material in this file were available by contacting Dr. Mei Li, li_mei76@163.com.
